# Supplementary material for: Effects of COVID-19 on trade flows: Measuring their impact through government policy responses
Source: PLoS One. 2021 Oct 13;16(10):e0258356. doi: 10.1371/journal.pone.0258356 (PMC8513914; doi:10.1371/journal.pone.0258356)
Supplement: S1 Table — High-income countries in bold. (DOCX) [file pone.0258356.s001.docx]

## S1 Table. List of exporting countries

| **Antigua and Barbuda** | **Germany** | North Macedonia |
| --- | --- | --- |
| Armenia | **Greece** | Pakistan |
| **Australia** | Guatemala | Paraguay |
| Azerbaijan | Guyana | Peru |
| Barbados | Hong Kong | Philippines |
| Belarus | Hungary | **Poland** |
| **Belgium** | **Iceland** | **Portugal** |
| Belize | **Ireland** | Romania |
| Brazil | **Israel** | Rwanda |
| Bulgaria | **Japan** | Senegal |
| **Canada** | Kyrgyzstan | Serbia |
| **Chile** | **Latvia** | **Slovakia** |
| Colombia | **Lithuania** | **Slovenia** |
| Croatia | **Luxembourg** | South Africa |
| **Cyprus** | **Macao** | **Spain** |
| **Czech Republic** | Madagascar | **Sweden** |
| **Denmark** | **Mauritius** | Turkey |
| Ecuador | Mexico | Ukraine |
| Egypt | Moldova | **United Kingdom** |
| El Salvador | Montenegro | Uzbekistan |
| **Estonia** | **Morocco** | Zambia |
| **Finland** | **Netherlands** | Zimbabwe |
| Georgia | **New Zealand** | North Macedonia |

Note: High-income countries in bold.
