# Supplementary material for: Effects of COVID-19 on trade flows: Measuring their impact through government policy responses
Source: PLoS One. 2021 Oct 13;16(10):e0258356. doi: 10.1371/journal.pone.0258356 (PMC8513914; doi:10.1371/journal.pone.0258356)
Supplement: S2 Table — (DOCX) [file pone.0258356.s002.docx]

## S2 Table. Main descriptive statistics

| **Variable** | **Mean** | **Std. Dev.** | **Min** | **Max** |
| --- | --- | --- | --- | --- |
| **Exports (million $)** | 53.897 | 444.267 | 0.000 | 31,152.740 |
| **COVID-19 shock** | 0.364 | 0.481 | 0.000 | 1.000 |
| **Stringency index** | 21.453 | 30.117 | 0.000 | 100.000 |
| **Economic Support index** | 22.405 | 34.375 | 0.000 | 100.000 |
| **Containment and Health index** | 19.982 | 27.245 | 0.000 | 85.011 |
| **Government Response index** | 20.301 | 27.645 | 0.000 | 82.420 |
| **Distance (in kilometres)** | 6,692.202 | 4,443.625 | 59.617 | 19,772.340 |
| **Contiguity** | 0.025 | 0.156 | 0.000 | 1.000 |
| **Common Language** | 0.133 | 0.340 | 0.000 | 1.000 |
| **Colonial Linkage** | 0.051 | 0.220 | 0.000 | 1.000 |
| **Regional Trade Agreement** | 0.421 | 0.494 | 0.000 | 1.000 |
