# Supplementary material for: Effects of COVID-19 on trade flows: Measuring their impact through government policy responses
Source: PLoS One. 2021 Oct 13;16(10):e0258356. doi: 10.1371/journal.pone.0258356 (PMC8513914; doi:10.1371/journal.pone.0258356)
Supplement: S3 Table — Dependent variable is trade flows. Robust standard errors in parentheses, such as *** p<0.01, ** p<0.05, * p<0.1. All the specifications include exporter-month, importer-month and pair fixed effects. (DOCX) [file pone.0258356.s003.docx]

## S3 Table. Results with one lag of COVID-19 government response indicators estimated by PPML, January 2019–October 2020

| **Column** | **(I)** | **(II)** | **(III)** | **(IV)** | **(V)** |
| --- | --- | --- | --- | --- | --- |
| **Dependent variable** | **Exports** | **Exports** | **Exports** | **Exports** | **Exports** |
| **COVID-19 shock** | -0.067*** |  |  |  |  |
|  | (0.016) |  |  |  |  |
| **Stringency * RTA** |  | -0.012*** |  |  |  |
|  |  | (0.004) |  |  |  |
| **Economic Support * RTA** |  |  | -0.014*** |  |  |
|  |  |  | (0.004) |  |  |
| **Containment and Health * RTA** |  |  |  | -0.012*** |  |
|  |  |  |  | (0.004) |  |
| **Government Response * RTA** |  |  |  |  | -0.012*** |
|  |  |  |  |  | (0.004) |
| **Constant** | 6.862*** | 6.859*** | 6.860*** | 6.859*** | 6.860*** |
|  | (0.004) | (0.004) | (0.004) | (0.004) | (0.004) |
| **Observations** | 160,208 | 160,208 | 160,208 | 160,208 | 160,208 |
| **Pseudo R2** | 0.985 | 0.985 | 0.985 | 0.985 | 0.985 |

*Notes: Dependent variable is trade flows. Robust standard errors in parentheses, such as *** p<0.01, ** p<0.05, * p<0.1. All the specifications include exporter-month, importer-month and pair fixed effects.*
