# Supplementary material for: Effects of COVID-19 on trade flows: Measuring their impact through government policy responses
Source: PLoS One. 2021 Oct 13;16(10):e0258356. doi: 10.1371/journal.pone.0258356 (PMC8513914; doi:10.1371/journal.pone.0258356)
Supplement: S4 Table — Robust standard errors in parentheses, such as *** p<0.01, ** p<0.05, * p<0.1. All the specifications include exporter-month, importer-month and pair fixed effects. (DOCX) [file pone.0258356.s004.docx]

## S4 Table. Robustness: Imports, *M*. Results by COVID-19 government response indicator estimated by PPML, January 2019–October 2020

| **Column** | **(I)** | **(II)** | **(III)** | **(IV)** | **(V)** |
| --- | --- | --- | --- | --- | --- |
| **Dependent variable** | **Imports** | **Imports** | **Imports** | **Imports** | **Imports** |
| **COVID-19 shock** | -0.045*** |  |  |  |  |
|  | (0.010) |  |  |  |  |
| **Stringency * RTA** |  | -0.014*** |  |  |  |
|  |  | (0.002) |  |  |  |
| **Economic Support * RTA** |  |  | -0.016*** |  |  |
|  |  |  | (0.002) |  |  |
| **Containment and Health * RTA** |  |  |  | -0.014*** |  |
|  |  |  |  | (0.002) |  |
| **Government Response * RTA** |  |  |  |  | -0.014*** |
|  |  |  |  |  | (0.002) |
| **Constant** | 20.702*** | 20.705*** | 20.706*** | 20.705*** | 20.706*** |
|  | (0.002) | (0.003) | (0.002) | (0.003) | (0.003) |
| **Observations** | 144,120 | 144,120 | 144,120 | 144,120 | 144,120 |
| **Pseudo R2** | 0.993 | 0.993 | 0.993 | 0.993 | 0.993 |

*Notes: Robust standard errors in parentheses, such as *** p<0.01, ** p<0.05, * p<0.1. All the specifications include exporter-month, importer-month and pair fixed effects.*

º
