# Supplementary material for: Effects of COVID-19 on trade flows: Measuring their impact through government policy responses
Source: PLoS One. 2021 Oct 13;16(10):e0258356. doi: 10.1371/journal.pone.0258356 (PMC8513914; doi:10.1371/journal.pone.0258356)
Supplement: S5 Table — Results by income levels. Estimated by PPML, January 2019–October 2020. Dependent variable is exports. Robust standard errors in parentheses, such as *** p<0.01, ** p<0.05, * p<0.1. Each COVID-19 indicator is estimated on a different regression but paired in the same column for the sake of brevity. All the specifications include exporter-month, importer-month and pair fixed effects. (DOCX) [file pone.0258356.s005.docx]

## S5 Table. One lag of COVID-19 government response indicator. Results by income levels. Estimated by PPML, January 2019–October 2020

|  | **Within groups** | | **Between groups** | |
| --- | --- | --- | --- | --- |
| **Column** | **(I)** | **(II)** | **(III)** | **(IV)** |
| **Exporter** | **High** | **Low** | **High** | **Low** |
| **Importer** | **High** | **Low** | **Low** | **High** |
| **COVID-19 shock** | -0.102*** | -0.087*** | 0.014 | 0.025 |
|  | (0.038) | (0.018) | (0.017) | (0.029) |
| **Stringency * RTA** | -0.027*** | -0.013*** | 0.003 | 0.009 |
|  | (0.010) | (0.004) | (0.004) | (0.007) |
| **Economic Support * RTA** | -0.024*** | -0.013*** | 0.003 | -0.002 |
|  | (0.009) | (0.005) | (0.004) | (0.009) |
| **Containment and Health * RTA** | -0.026*** | -0.013*** | 0.003 | 0.009 |
|  | (0.010) | (0.004) | (0.004) | (0.007) |
| **Government Response * RTA** | -0.026*** | -0.013*** | 0.002 | 0.008 |
|  | (0.010) | (0.004) | (0.004) | (0.007) |
| **Observations** | 21,652 | 45,955 | 72,914 | 19,479 |

*Notes: Dependent variable is exports. Robust standard errors in parentheses, such as *** p<0.01, ** p<0.05, * p<0.1. Each COVID-19 indicator is estimated on a different regression but paired in the same column for the sake of brevity. All the specifications include exporter-month, importer-month and pair fixed effects.*
