# Supplementary material for: Effects of COVID-19 on trade flows: Measuring their impact through government policy responses
Source: PLoS One. 2021 Oct 13;16(10):e0258356. doi: 10.1371/journal.pone.0258356 (PMC8513914; doi:10.1371/journal.pone.0258356)
Supplement: S6 Table — Dependent variable is imports. Robust standard errors in parentheses, such as *** p<0.01, ** p<0.05, * p<0.1. Each COVID-19 indicator is estimated on a different regression but paired in the same column for the sake of brevity. All the specifications include exporter-month, importer-month and pair fixed effects. (DOCX) [file pone.0258356.s006.docx]

## S6 Table. Robustness: Imports, *M*. Results by income levels and COVID-19 indicators estimated by PPML, January 2019-October 2020

|  | **Within groups** | | **Between groups** | |
| --- | --- | --- | --- | --- |
| **Column** | **(I)** | **(II)** | **(III)** | **(IV)** |
| **Importer** | **High** | **Low** | **High** | **Low** |
| **Exporter** | **High** | **Low** | **Low** | **High** |
| **Dependent variable** | $\boldsymbol{M}_{\boldsymbol{ijm}}$ | $\boldsymbol{M}_{\boldsymbol{ijm}}$ | $\boldsymbol{M}_{\boldsymbol{ijm}}$ | $\boldsymbol{M}_{\boldsymbol{ijm}}$ |
| **COVID-19 shock** | **-0.086***** | **-0.023** | **-0.057***** | **0.067***** |
|  | **(0.031)** | **(0.014)** | **(0.016)** | **(0.022)** |
| **Stringency * RTA** | **-0.028***** | **-0.013***** | **-0.012***** | **0.021***** |
|  | **(0.008)** | **(0.003)** | **(0.004)** | **(0.006)** |
| **Economic Support * RTA** | **-0.027***** | **-0.011***** | **-0.013***** | **0.016**** |
|  | **(0.008)** | **(0.004)** | **(0.004)** | **(0.008)** |
| **Containment and Health * RTA** | **-0.026***** | **-0.014***** | **-0.011**** | **0.022***** |
|  | **(0.008)** | **(0.003)** | **(0.004)** | **(0.006)** |
| **Government Response * RTA** | **-0.026***** | **-0.014***** | **-0.011***** | **0.022***** |
|  | **(0.008)** | **(0.003)** | **(0.004)** | **(0.006)** |
| **Observations** | **22,282** | **37,967** | **63,456** | **20,071** |

***Notes: Dependent variable i****s imports. Robust standard errors in parentheses, such as *** p<0.01, ** p<0.05, * p<0.1. Each COVID-19 indicator is estimated on a different regression but paired in the same column for the sake of brevity. All the specifications include exporter-month, importer-month and pair fixed effects.*
