# Supplementary material for: Effects of COVID-19 on trade flows: Measuring their impact through government policy responses
Source: PLoS One. 2021 Oct 13;16(10):e0258356. doi: 10.1371/journal.pone.0258356 (PMC8513914; doi:10.1371/journal.pone.0258356)
Supplement: Attachment — Submitted filename: Response to Reviewers.docx [file pone.0258356.s007.docx]

Letter to editor

Responses to Editor and Reviewer's Reports on PONE-D-21-12117, “Effects of COVID-19 on trade flows: measuring their impact through government policy responses”

We would like to thank the Editor for offering us the opportunity to revise and resubmit our manuscript to PLOS ONE and for your interest, effort and comments on our manuscript.

All comments were very well taken, and we have tried to address them as best as

we could in preparing the revised version of the manuscript.

We organize our responses to reviewers in the same order as comments appeared in the editor letter. Further, for your convenience, we repeat comments in blue color before describing how we dealt with each of them

We have substantially revised the paper based on the comments and suggestions by the two reviewers and the Editor. In sum, in our view, the revision has led to a substantially improved paper.

Let us summarize the main changes in the paper:

1. We have extended the discussion.
2. We have revised the structure of the paper.
3. We have updated the literature incorporating those reference that appeared since we send the paper to review, note that the topic is burgeon.
4. We fixed the format of the document (references, figures…)

These updates have substantially improved the content of the document and we thank for it. We hope you find the revision satisfactory.

Sincerely,

**Editor Comments**

**Comment:** “The discussion should be extended, whereas the structure of the paper should be revised.”

**Authors´ response:** We have done both, extend the discussions and revise the structure.

We have widely extended the discussion mainly in section 2 “Literature review on COVID-19 and trade” and in the new section entitle “Policy responses to COVID-19: equal or unequal?” but we also have new paragraphs in sections “Results by levels of economic development” and “Conclusions” These new content focus on the policy response of government explaining better the COVID-19 related measures, in which the paper focus. We also guide the readers to additional sources of information on policy measures adopted by countries from a more general economic policy perspective (monetary, fiscal, etc.), of lower relevance in the paper.

We have a new section “Empirical analysis” At the beginning of this section we explain “This section is organized into three separate sub-sections. First, we describe the empirical model and the estimation strategy. Second, we report information on data issues. Finally, we cover country policy responses to COVID-19.” The new section merges previous sections: “Empirical model” and “Data and estimation strategy” extending content with additional information.

**Reviewer 1 comments**

**Comment 1:** “It would be better for the authors to give the readers more information about what policy or steps are taken during different time period. Please explain more about the different trends in Figure 1 and Figure 2.”

**Comment 3:** “Although the author compared countries with different income levels, we still have no idea about which countries really suffered in the COVID-19 and what are the differences between government policy responses. More discussion or insights should be given here.”

**Authors´ response to both comments:** Thank you for the comments, the paper is now more informative on economic policy responses. We have included additional information. First, we have redesign Figure 1 providing additional detail for countries and confidence intervals for the different indicators, we introduce explanations of the new Figure 1; for harmonization purposes we also modified figure 2. We copy here the new figure 1:

**Figure 1. Evolution of the four COVID-19 policy indicators between December 2019 and October 2020 by income level for our sample of exporting countries.** Source: own elaboration from [1]. Note: each point represents a country, and the concentration of countries with similar values produces darker areas. Additionally, the mean and 95% confidence bands are represented.

Second, we pay special attention to differences between high-income and low- and middle-income countries and the set of policy indicators developed by Hale et al. (2021), the most remarkable approach to measure countries´ policy responses, in line with our research objectives. Third, we suggest additional references for the interested reader in the analysis of differences between government policy responses. Apart from less noticeable changes we highly the following paragraphs:

We acknowledge that policy responses differ by country, as the impacts of COVID-19 have been strongly unequal for countries due to several reasons. First, countries have reported differences in the number of deaths, mainly attributable to the population composition. There is an increasing number of elder populations in a significant number of high-income OECD countries [2] and this group is the most vulnerable to COVID-19 (e.g., [3]).

At the same time, countries have also implemented trade policy actions to mitigate the influence of COVID-19 on the global economy^[1]^. [4] summarizes the major stylized facts during the first nine months of the pandemic^[2]^. First, there was a noticeable rise in trade policy activism consisting mainly of export controls and import liberalization measures with strong cross-country variation. Second, this activism was reported to vary by country and products, where medical and food products experimented a substantial overall increase in their demand from February 2020. Third, we observe a further trade liberalization process after May 2020, where the number of liberalization measures exceeded the number of trade restrictions in medical products.

Such cross-country variation in trade policy response aligns with our expectations since, as mentioned previously, trade specialization differs by country. Accordingly, their sensitivity to the growing demand for food and medical products may vary substantially. For this reason, some countries were more resilient to COVID-19 trade shocks than other countries, as shown by the decreases observed in their trade flows. To this end, we compare trade drops for the most affected countries relative to January 2020 and their governmental response, from May 2020 to October 2020. As described by [4], countries experienced a substantial relaxation in most of their trade measures in May 2020.

For the ten countries with the largest trade drop evidence is ambivalent ^[3]^. On the one hand, four high-income (Macao, Mauritius, Portugal, and Slovakia) and six middle-income countries (El Salvador, Mexico, Montenegro, Guyana, Egypt, and Romania) were among the most affected countries in May 2020, with El Salvador registering the highest level of governmental response. Trade relative to January 2020 ranges from 51 to 69 percent in this period. On the other hand, we find that the number of high-income countries increased to six in October 2020 but, at the same time, differences in governmental response decreased their observed variance. Israel registered the highest level of governmental response during this month. In this case, relative trade ranges from 76 to 102, corroborating the previous finding that countries recovered rapidly from this trade shock. To sum up, despite differences in governmental response due to the impact of COVID-19 by countries, recovery can be alleged to follow similar patterns for the most affected countries.

We consider that with the modifications we provide more insights about differences between government policy responses and income levels.

**Comment 2:** “Pay attention to the structure of your paper. Maybe the estimation strategy should be organized in the section of model description.”

**Authors´ response:** We have rearranged sections and subsections in the manuscript: we have moved data and estimation strategy to section 3, which has been renamed to “empirical analysis”. Estimation strategy has been merged with empirical model, in a new sub-section entitled “Model description and estimation strategy”. With this change, manuscript structure is more streamlined, and its soundness has also increased in comparison to the previous version.

**Reviewer 2**

**Comment 1:** “This paper examines the impact of COVID-19 on bilateral trade flows using a gravity model of trade. The empirical analysis is designed properly. Using the monthly trade data of 68 countries, findings suggest a greater negative impact of COVID-19 on bilateral trade for countries that were members of regional trade agreements before the pandemic. The impact of COVID-19 is negative when governmental actions are considered. The article is well written.”

**Authors´ response:** We thank the reviewer for comments and effort reviewing the manuscript.

References

1. Hale T, Angrist N, Goldszmidt R, Kira B, Petherick A, Phillips T, et al. A global panel database of pandemic policies (Oxford COVID-19 Government Response Tracker). Nature human behaviour. 2021;5: 529-538. doi: <https://doi.org/10.1038/s41562-021-01079-8>.

2. OECD. Elderly population. . 2014. doi: <https://doi.org/https://doi.org/10.1787/8d805ea1-en>.

3. Daoust J. Elderly people and responses to COVID-19 in 27 Countries. PloS one. 2020;15. doi: <https://doi.org/10.1371/journal.pone.0235590>.

4. Evenett S, Fiorini M, Fritz J, Hoekman B, Lukaszuk P, Rocha N, et al. Trade policy responses to the COVID‐19 pandemic crisis: Evidence from a new data set. The World Economy. 2021. doi: <https://doi.org/10.1111/twec.13119>.

stylefix

1. For the sake of brevity and in line with the aim of the article, we only consider the trade policy response. Readers interested in analyzing a complete set of economic policy responses (i.e., budgetary and/or monetary) to COVID-19 are entitled to check the International Monetary Fund (IMF) Policy Tracker at <https://www.imf.org/en/Topics/imf-and-covid19/Policy-Responses-to-COVID-19>
2. We focus on the corresponding period coincident with our sample, but further trade policy actions have been implemented after this period because of the increasing number of cases related to COVID-19 in subsequent waves. They can be checked at the IMF Policy Tracker previously reported, which is updated regularly.
3. For the sake of brevity, we have omitted the data concerning each country and provide a general overview. Data is available from the authors upon request to the corresponding author and can be obtained from the UN Comtrade database.
